# Wide-Awake Tenolysis of a Pectoralis Major to Biceps Transfer After Near Total Arm Avulsion Amputation

**DOI:** 10.7759/cureus.28837

**Published:** 2022-09-06

**Authors:** Ajeesh Sankaran, K R Thushara, V Ajaykumar, E G Mohankumar

**Affiliations:** 1 Hand and Reconstructive Microsurgery, Kerala Institute of Medical Sciences (KIMS) Al Shifa Super Speciality Hospital, Perinthalmanna, IND; 2 Orthopaedic Surgery, Kerala Institute of Medical Sciences (KIMS) Al Shifa Super Speciality Hospital, Perinthalmanna, IND

**Keywords:** tenolysis, wide awake hand surgery, tendon transfer, biceps brachii, pectoralis major

## Abstract

Wide-awake surgery is transforming many areas of hand surgery. We report a distinctive case of an avulsion near total amputation of the right dominant arm, which required emergent shaft humerus fracture fixation and brachial artery repair with a vein graft. Three months post-injury, the patient underwent long segment nerve grafts of the median and ulnar nerves, with pectoralis major to biceps transfer for elbow flexion reconstruction. Since the patient failed to gain any functional movement of the elbow, we explored the transfer under wide-awake local anaesthesia using lignocaine and adrenaline. Four months after the wide-awake release, the patient had gained 70 degrees elbow flexion against gravity and 110 degrees with gravity eliminated. On the Waikakul scale, the result was categorized as 'Good'. Wide-awake anaesthesia allowed sufficient release of a large muscle transfer in a prior traumatised zone with a satisfactory result.

## Introduction

Wide-awake hand surgery is an emerging concept and is fast gaining broad acceptance. The safe use of lignocaine with adrenaline mixture for anaesthesia of the digit, once considered abhorrent, is a basic premise for its application [1,2]. This idea has since been applied to more complex procedures in more proximal locations of the limbs [3,4]. We report one such instance: tenolysis of a pectoralis major to biceps transfer, in an arm with an underlying vein graft. The case illustrates how the wide-awake approach helped solve a complex clinical issue successfully.

## Case presentation

**Figure 1:**
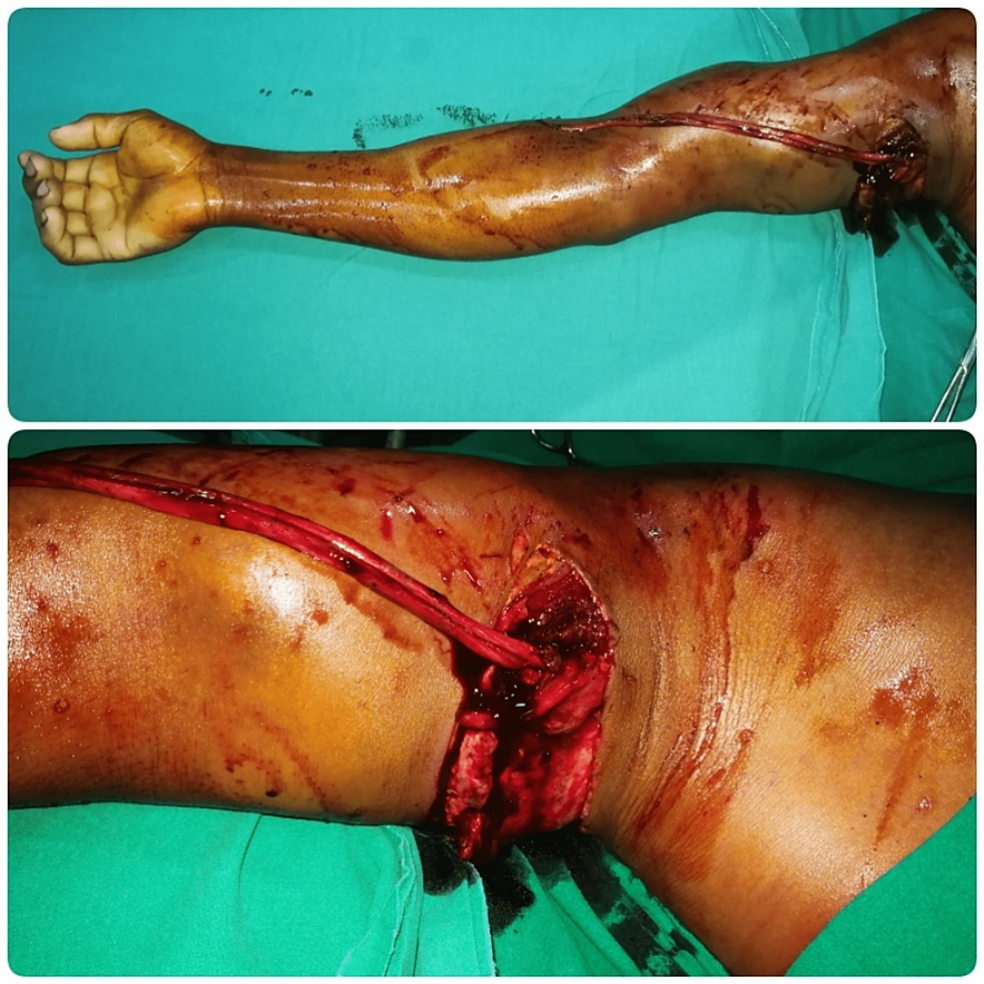
Near total amputation of the right arm – injury pictures

**Figure 2:**
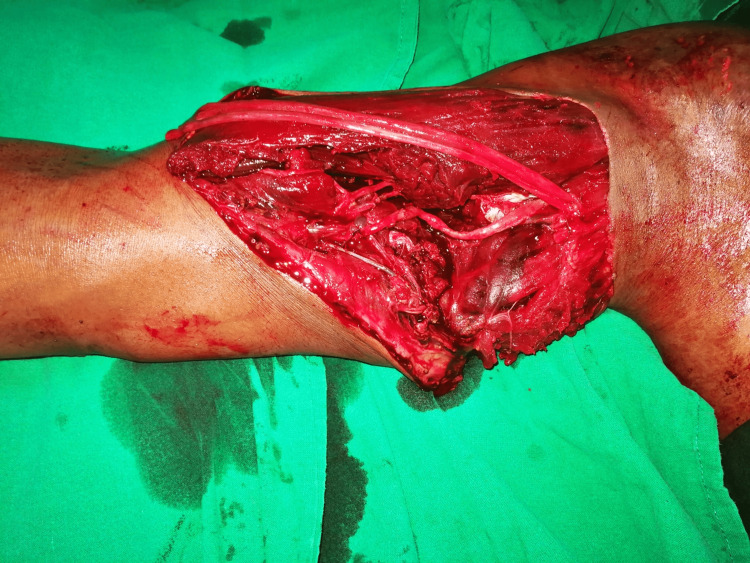
Post fracture fixation and reversed saphenous vein grafting. Avulsed median and ulnar nerves visible

A 28-year-old man suffered a conveyor belt avulsion injury of his right dominant upper limb. He presented with a near-total amputation of the arm at the mid-humerus level, with only the anterior and lateral skin with subcutaneous tissue being intact (Figure 1). After initial resuscitation, he underwent emergent humerus fracture fixation by plating and brachial artery reconstruction with a reversed saphenous vein graft (Figure 2). Exploration also revealed a complete infraclavicular brachial plexus injury, which could not be reconstructed primarily due to the avulsion nature of the injury. The median and ulnar nerves were tagged subcutaneously for future reconstruction [5]. The musculocutaneous nerve was found to be similarly severely injured, along with muscle loss of the biceps and the brachialis. The triceps belly was found to be injured and repaired, while the radial nerve was confirmed to be avulsed as well. After surgery, the limb survived uneventfully and a posteromedial arm residual wound was covered with a split skin graft four days after the injury.

**Figure 3:**
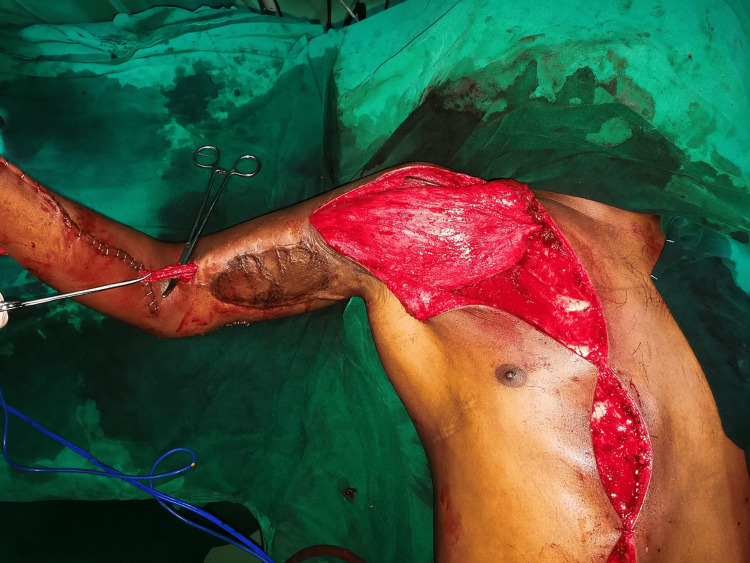
Bipolar transfer of the pectoralis major to the biceps

The patient was immobilized in an above elbow splint for four weeks, after which shoulder strengthening and passive mobilization of other joints were instituted. Reconstruction for the infra-clavicular injury was planned, including a tendon transfer for elbow flexion. The latissimus dorsi transfer for the same was ruled out since the transfer would have to be routed through the prior traumatised zone of the arm. Hence, the pectoralis major was chosen as the donor and isolation exercises began one month after the injury. At three months post-injury, the patient underwent a bipolar transfer of the pectoralis major to the biceps tendon (Figure 3). The proximal end was fixed to the lateral clavicle by trans-osseous sutures and the muscle belly tunnelled via the anterior arm, avoiding the skin grafted area. Suturing was performed to the distal biceps tendon with the shoulder adducted and flexed forward by 60 degrees, and with the elbow in 120 degrees flexion. The resulting muscle tension allowed the elbow to maintain 90 degrees flexion spontaneously. The median and ulnar nerves were reconstructed with long segment grafts harvested from both the sural nerves, the ipsilateral superficial radial nerve, and the ipsilateral lateral forearm cutaneous nerve. Post-operatively, the elbow was immobilized in 90 degrees flexion, and mobilization was begun at six weeks in a custom brace. Initially, full flexion was allowed from a position of 60 degrees flexion. Extension was progressively increased to reach full extension by 12 weeks and strengthening continued thereafter. By four months post transfer, strong contraction of the pectoralis major could be felt in the shoulder region. However, only minimal elbow movement could be discerned with gravity eliminated and no antigravity flexion was possible. After discussion with the patient, it was decided to go forward with an exploration of the transfer zone. Specifically, wide-awake release was offered for intra-operative active mobilization to verify the adequacy of the release.

**Figure 4:**
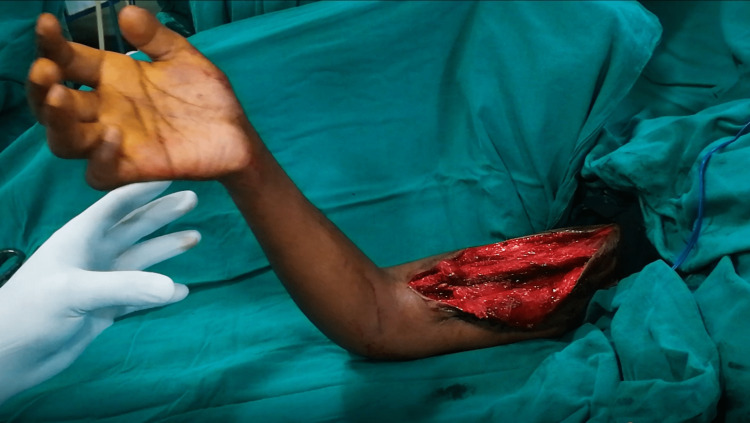
Intra-operative elbow flexion after wide-awake tenolysis

The release was performed without any sedation, but with the anaesthesia team on standby. At our centre, we have routinely used premixed two percent lignocaine and 1:100,000 adrenaline solution, diluted to half strength. For this patient, 40 ml of such a solution was prepared and buffered with 4ml of 8.4% sodium bicarbonate to decrease the pain of the injection. The solution was infiltrated diffusely over the anterior arm region, followed by a waiting period of half an hour for optimal hemostatic activity of the adrenaline component. The area of the tunnelled pectoralis major was exposed and the adhesions were released sequentially (Video 1). We found that adhesions were mostly in the region adjacent to the skin graft. It was also realized that the transfer was slightly loose, which was remedied by tightening the transfer. The patient was able to achieve 90^o^ antigravity elbow flexion intra-operatively (Figure 4, Video 1).

**Video 1:** Perioperative video of the tenolysis

**Figure 5:**
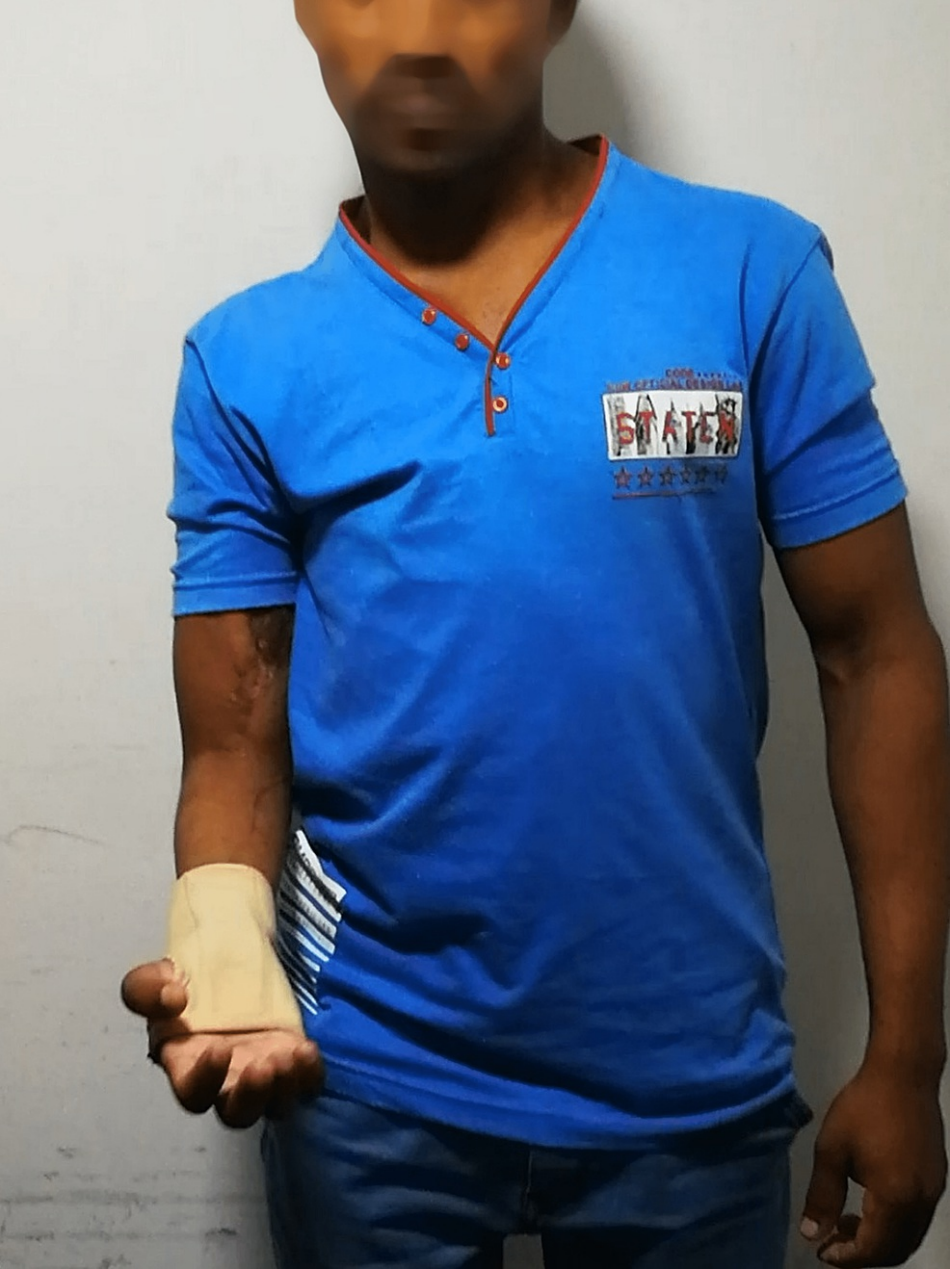
Follow-up at four months after release

Four months post-release the patient had achieved 70 degrees antigravity elbow flexion with a flexion contracture of 15 degrees. He had near full flexion with the shoulder abducted and gravity eliminated (110 degrees), (Figure 5). He had better than M3 power, which was also rated as a ‘Good’ result by the scheme described by Waikakul et al. [6].

## Discussion

We report the successful release of a proximal tendon transfer under local anaesthesia, in a region with an underlying critical vascular reconstruction. In our department, wide-awake anaesthesia is routinely used for flexor tendon injuries (Zones I - III), extensor injuries (Zones I - VII), and tenolysis. Armed with this experience, the above procedure was planned under local anaesthesia to help diagnose the reason for the lack of active motion, rectify it, and confirm the attainment of functional motion.

Wide-awake hand surgery, often also termed wide-awake local anaesthesia no tourniquet (WALANT), is now established as a reliable and safe technique for multiple hand procedures [1,3,4,7]. Spurred by these successes, now the same techniques have been extended further afield to fractures of the distal radius [4,7], olecranon [8], and clavicle [9]. In the lower limb, many foot procedures have also been reported to be similarly managed [10]. While tenolysis in the hand with WALANT techniques is well established [11], the above case remains unique in its application of local anaesthesia for an upper arm level tenolysis.

The original reluctance for the use of adrenaline in digital anaesthesia was, of course, due to a perceived risk of gangrene. The sheer number of cases for which adrenaline has been used safely now demonstrates otherwise [12,13]. But administering adrenaline in an area with a prior repaired vascular injury is certainly intimidating. Lu et al. have reported a near-total wrist amputation which later underwent correction of a tendon repair mismatch and tenolysis under WALANT [14]. The successful use of adrenaline along with local anaesthesia for digit replantation/ revascularization has also been reported [15]. While we noticed no changes in the vascularity of the limb after the diffuse infiltration of adrenaline close to a critical vein graft, a phentolamine rescue solution was kept ready. It is also worth noting that the use of adrenaline may not be as perilous in the arm as in the digit, due to the presence of collaterals at the arm level. However, given the nature of the initial injury in this patient, such collaterals may very well not be sufficient for limb survival.

Patient perception of wide-awake hand surgery has been reported to be positive [16,17]. Involving patients in their care can be seen as a first step in ensuring post-operative compliance. Since our patient had suffered such a devastating injury with the function of the entire upper limb at stake, the psychological impact of seeing the elbow move at the time of surgery cannot be understated. In addition, the wide-awake approach also allowed us to identify the laxity in the transfer which would certainly have been difficult under regional or general anaesthesia.

## Conclusions

The reported case illustrates that the use of adrenaline with lignocaine in a revascularized limb can be safe and effective. The wide-awake approach allowed the identification of problems (such as laxity of the transfer) that could not have been recognized and solved otherwise. Difficult clinical problems require the use of non-conventional ideas to achieve the best possible outcomes.
